# microRNA as an Important Mediator in the Regulation of Male *Gallus gallus domesticus* Reproduction: Current State of the Problem

**DOI:** 10.3390/ijms26010112

**Published:** 2024-12-26

**Authors:** Marina Pozovnikova, Anastasiya Ivershina, Olga Stanishevskaya, Yuliya Silyukova

**Affiliations:** Russian Research Institute of Farm Animal Genetics and Breeding—Branch of the L.K. Ernst Federal Research Center for Animal Husbandry, Pushkin, Saint-Petersburg 196625, Russia; pozovnikova@gmail.com (M.P.); olgastan@list.ru (O.S.)

**Keywords:** gene expression, germ cells, epigenetic factors, transcriptome, microRNA biogenesis, sperm, gonads, rooster

## Abstract

During all periods of male ontogenesis, physiological processes responsible for the correct functioning of reproductive organs and spermatogenesis are under the influence of various factors (neuro-humoral, genetic, and paratypical). Recently, the attention of researchers has increasingly turned to the study of epigenetic factors. In scientific publications, one can increasingly find references to the direct role of microRNAs, small non-coding RNAs involved in post-transcriptional regulation of gene expression, in the processes of development and functioning of reproductive organs. Although the role of microRNAs in the reproduction of mammals, including humans, has been intensively studied, this area of knowledge in birds remains under-researched and limited to single experiments. This is likely due to the unique features of embryogenesis and the structure of the avian reproductive system. This review summarizes the current state of knowledge on the role of microRNAs in avian reproduction. Insight into the molecular basis of spermatogenesis in *Gallus gallus domesticus* is provided. Data on the functions and mechanisms by which microRNAs influence the processes of growth, development, and formation of rooster germ cells that determine the necessary morphofunctional qualitative characteristics of mature spermatozoa are summarized. Particular attention is paid to miRNA biogenesis as an important step affecting the success of spermatogenesis, as well as the role of miRNAs in avian sex differentiation during early embryogenesis. The modern literature sources systematized in this review, revealing the questions about the role of miRNAs in the reproductive function of birds, create a theoretical basis and define new perspectives and directions for further research in this field.

## 1. Introduction

Male fertility is an important aspect of reproductive biology that determines the ability of male individuals to pass on their genes to the next generation, which is essential for both biodiversity conservation and the production of healthy offspring. The reproductive potential of males is largely determined during early ontogenesis. The development of gonads is the crucial first step in sexual differentiation that subsequently leads to the establishment of hormonal, phenotypic, neurological, and behavioral differences. This step is significantly affected by changes in the hormonal environment of the embryo [1]. The process of sperm development, known as spermatogenesis, begins at puberty in males and continues throughout life. It occurs in the seminiferous tubules of the testis and consists of several stages, from the division of spermatogonia to the formation of mature spermatozoa. Spermatogenesis includes the phases of mitosis, meiosis, and the subsequent maturation of germ cells [2,3,4]. Male fertility is determined by the presence of adequately matured gametes, spermatozoa, and the variability of fertility is related to their number in the ejaculate and morphofunctional properties.

**Figure 1:**
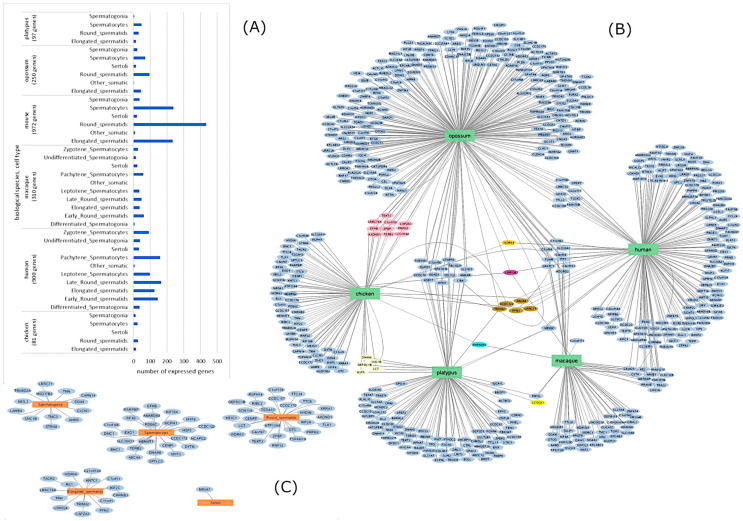
Number of expressed genes in germ and somatic cells of the male reproductive organs in mammals and birds (adapted from Murat, F. et al., 2023 [12]) (**A**). The *x* axis contains data on the number of expressed genes, the *y* axis contains data on the species and cell type. The diagram is divided into clusters by species, number of expressed genes and cell types that were included in the analysis. Each bar represents the number of expressed genes for each cell type. The network of genes expressed in germ and somatic cells for each species (**B**). Central gene clusters are formed by genes whose expression is observed in several species, and the lines lead to the species themselves. The pink cluster contains genes shared by opossum and chicken, yellow cluster—by human, opossum and chicken, bright yellow—by platypus and macaque, turquoise cluster—by chicken and macaque, purple—by chicken, human, macaque, opossum, and the brown cluster—by chicken, human, platypus and opossum. Clusters of genes expressed in roosters by cell type (**C**). Gene and microRNA networks were visualized using the https://cytoscape.org/ (accessed on 2 December 2024) platform.

Throughout all periods of male ontogenesis, physiological processes that ensure proper functioning of reproductive organs, as well as the process of spermatogenesis, are influenced by paratypical (feed ration, housing system, climate) and physiological (hormones of the hypotholamic-pituitary-gonadal (HPG) axis, neuroregulation) factors that vary according to the specific characteristics of the individual. All these processes require precise temporal and spatial coordination, which is ensured by genetic and epigenetic components [5,6]. To date, a significant number of protein-coding genes that play key roles in spermatogenesis have been identified. These proteins are involved in processes such as cell division and differentiation, flagellar integrity and movement, mitochondrial function, sperm maturation and storage in the female tract, and oocyte-sperm interactions [7,8]. Whole transcriptome sequencing of testis and epididymis reveals genes associated with sperm development in roosters. Generally, each stage of spermatogenesis is characterized by transcriptomic changes in different gene groups, with both protein-coding and non-coding RNA (ncRNA) genes being expressed [9]. The majority of fertility-related genes exhibit conserved expression trajectories, with the number of these genes varying among some mammalian and avian species (Figure 1).

Gene expression is known to be regulated by multiple mechanisms, including transcriptional, posttranscriptional, and epigenetic processes. However, to date, these mechanisms have not been fully understood. Studies show that not only DNA sequence, but also epigenetic changes such as DNA methylation and histone modifications, can affect gene activity. These changes can be caused by external factors such as stress, nutrition, and even exposure to toxins, which emphasizes the importance of the environment to male fertility. Additionally, post-transcriptional mechanisms such as regulation of mRNA levels and processing also play an important role in controlling gene expression [10,11].

Recent advancements in RNA technologies have greatly enhanced the ability to identify and analyze the variety of transcripts found in semen. The examination of the germ cell transcriptomes, as well as somatic cell transcriptomes derived from reproductive organs of various male species, revealed some peculiarities that have been formed in the course of evolution. Nevertheless, during spermatogenesis in both mammals and birds, an increase in the expression of different types of ncRNAs and a decrease in protein-coding genes are observed after the meiosis stage [12]. The accumulation of ncRNA transcripts is associated with their crucial role in the regulation of gene expression at the transcriptional and post-transcriptional levels, which ultimately ensures the normal development of germ cells and their adaptation to various environmental conditions [13]. The latest data from the transcriptome of rooster testicular tissues obtained by next-generation sequencing (NGS) showed that one-third of the RNA transcripts of differentially overexpressed genes are ncRNAs [14].

Different patterns of ncRNA expression not only reflect the phases of spermatogenesis, but also play important roles during fertilization, early embryogenesis, and maintenance of normal embryo development. The ncRNAs are also involved in the paternal chromatin packaging, essential for the transfer of genetic information, and may influence sperm maturation and capacitation, which is important for successful fertilization. These molecules may also participate in intergenerational or transgenerational inheritance, modulating the genetic and phenotypic characteristics of the offspring [15].

The class of small ncRNAs includes miRNAs, which are key molecules involved in post-transcriptional gene regulation and play an important role in spermatogenesis in mammals and birds. They modulate the activity of specific protein-coding mRNAs responsible for germ cell development by affecting subtle regulatory mechanisms. Changes in the levels of miRNA expression at different stages of spermatogenesis, as a consequence, lead to changes in the concentration of key proteins required for proper implementation of the spermatogenesis program [16]. Recent studies show that disruption of miRNA expression can lead to abnormalities in spermatogenesis and, consequently, reduced fertility. Therefore, microRNAs are considered as promising biomarkers of fertility in mammals [17].

To date, the function and structure of miRNAs have been extensively studied in mammals. Various methods to detect and quantify microRNAs in tissues and organs are available, including northern blot, real-time quantitative PCR (qPCR), in situ hybridization (ISH), as well as microarray, miRNA expression analysis, and RNA sequencing [18]. Furthermore, specialized databases have been created to store and continuously update information on miRNAs from different species (http://www.mirbase.org; https://mirdb.org/index.html).

Although the role of miRNAs has been widely studied in reproductive aspects of mammals, including humans, this area of knowledge remains poorly understood in birds and is at the stage of individual experiments. Presumably, this may be related to the peculiarities of embryogenesis in birds (polylecithal oocyte, extrauterine development), as well as peculiarities of the male reproductive system (absence of accessory sex glands, intra-abdominal testes, and morphological features of spermatozoa—head shape, small volume of cytoplasm, and thin long flagellum). This challenges the application of standard research methods and approaches.

The aim of this overview was to summarize and discuss current advances in the study of the functional role and significance of miRNAs in the context of male avian reproduction.

## 2. *Gallus gallus domesticus* Spermatogenesis and Sperm RNA

**Figure 2:**
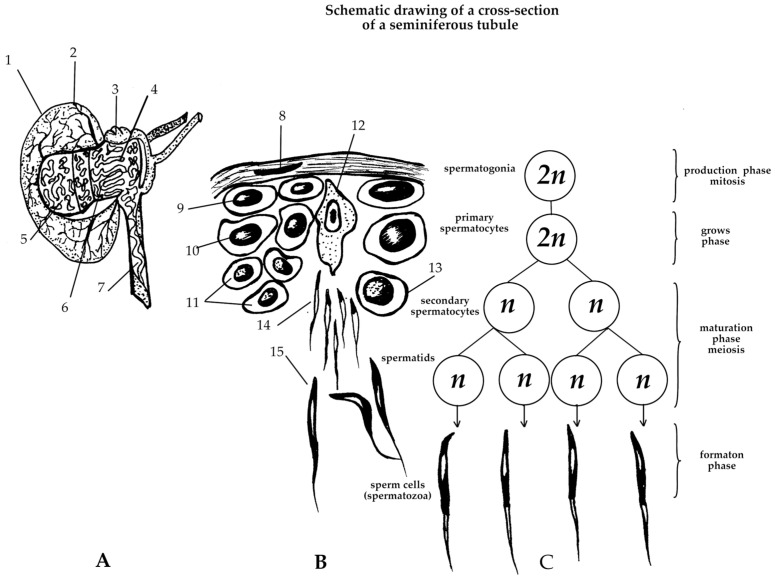
Scheme of a longitudinal cut of a rooster testis (**A**), a section of the seminiferous tubule (**B**), and spermatogenesis (**C**) in birds (adapted from Sturkie P.D. and Opel H., 1976 [19]). Cell development proceeds from the periphery to the center of the testis, where the network of ejaculatory ducts discharges the mature cells into the epididymis. 1—testis, 2—tunica albuginea, 3—rete tubules, 4—straight seminiferous tubules, 5—convoluted seminiferous tubules, 6—straight tubules, 7—sperm duct, 8—nucleus of the sheath cell of the seminiferous tubule, 9—spermatogonia, 10—1st order spermatocyte, 11—2nd order spermatocyte, 12—Sertoli cell, 13—early round spermatids, 14—late spermatids, 15—late elongated spermatids.

**Figure 3:**
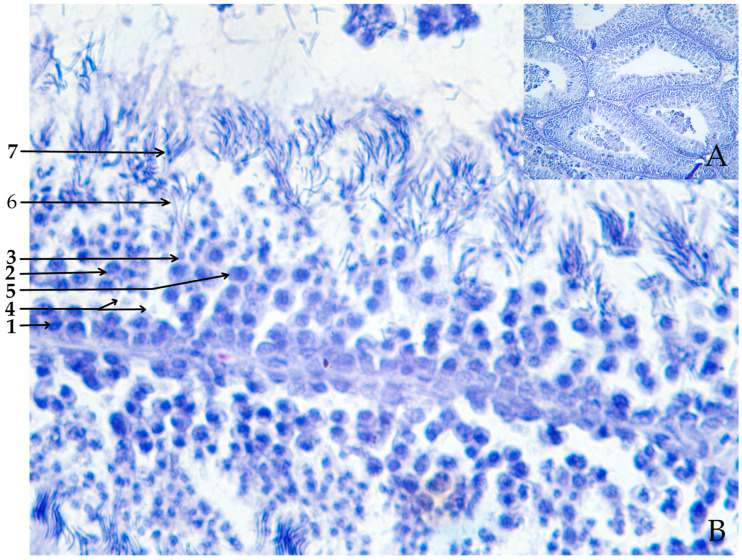
Fragment of histologic cross-section of rooster testis ((**A**) magnification ×200, (**B**) magnification ×400): 1—spermatogonia, 2—1st order spermatocyte, 3—2nd order spermatocyte, 4—Sertoli cell, 5—early round spermatids, 6—late spermatids, 7—late elongated spermatids.

Spermatogenesis (Figure 2 and Figure 3) occurs within the testes, namely in the seminiferous tubules, which have a complex structure and a large number of thin convoluted ducts. The seminiferous tubules in birds have an anastomosing structure and are surrounded by a basal lamina composed of fibroblasts, myoepithelial cells, and connective tissue. The epithelium of the tubule consists of Sertoli cells and spermatogenic cells, in which meiosis and further cell differentiation occurs. As a result, differentiated cells then migrate from the basal lamina into the tubule lumen. The Sertoli cells represent special sustencular cells that line the tubule, namely, they extend from the basal lamina of tubules to the lumen. Tight junctions between Sertoli cells divide the seminiferous tubule epithelium into basal and luminal compartments, creating an effective hematotesticular barrier. Sertoli cells play a key role in gamete development and maturation, and also perform an important endocrine function by producing the hormone inhibin, which determines spermatogonia proliferation and controls testosterone synthesis [19,20,21].

**Figure 4:**
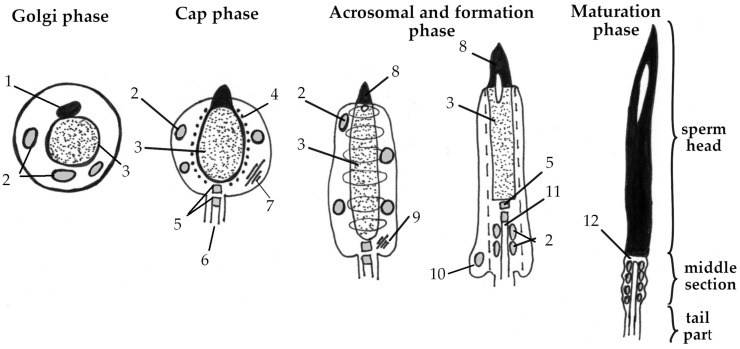
Scheme of spermatozoa formation in birds (adapted from Asano A. and Tajima A. (2017) [22]). Golgi phase—the beginning of head formation in spermatids; Cap phase—the development of the acrosomal cap; Acrosomal and formation phase—the elongation of the spermatid nucleus and the acquisition of specific traits; Maturation phase—the removal of excessive cytoplasm, final maturation. 1—acrosomal vesicle, 2— mitochondria, 3— nucleus, 4—microtubules, 5—centrioles, 6—developing flagellum; 7—Golgi apparatus, 8—acrosome, 9—remnants of the Golgi apparatus; 10—excess cytoplasm, 11—axoneme, 12—neck of the spermatozoon.

During embryogenesis, gonocytes develop from primordial germ cells (PGCs) and then spermatogonial stem cells (SSCs) develop from gonocytes in the testes. Finally, two daughter spermatogonia are developed from one SSC. The process of spermatogenesis begins with the spermatogonia mitosis, which provides enough cells for further division. The accumulation of a sufficient number of cells causes some of them to lose the ability to mitotic division, which means that they have differentiated into primary spermatocytes. Then, as a result of the first meiotic division, each primary spermatocyte undergoes a complex stage of chromosome reduction, transforming into two secondary spermatocytes. At this stage, each cell contains a haploid set of chromosomes, which is critical for maintaining genetic diversity. The secondary spermatocytes then undergo a second meiotic division and form spermatids. Spermatids are immature cells that have some degree of differentiation but still require additional changes to become a proper spermatozoon. Therefore, spermatids first lose excess cytoplasmic material and then the head, neck, and tail are formed (Figure 4). Mature spermatozoa are stored in the epididymis and ejaculatory ducts until ejaculation, when they are excreted through the genital tract [3,22]. The morphology of male germ cells changes from the moment of hatching until the age of 12 months. Mature spermatozoa are not identified until after 5 months of age [23].

Thus, during spermatogenesis, SSCs undergo a series of changes that result in the formation of mature spermatozoa capable of fertilization. Essentially, a spermatozoon is a unique cell that contains genetic material but is devoid of translational activity and is transcriptionally inert. At each stage of spermatogenesis during passage through epididymides, spermatozoa contact and accumulate various ncRNAs, transcripts, and proteins through the involvement of the mechanoenzyme dynamin 1 [24]. The spermatozoon contains both mature RNAs, which are presented by mRNAs that arise during spermatogenesis, and “foreign” mRNAs that are “acquired” during the passage through epididymis, as well as ncRNAs, which include fragmented and undegraded mRNAs, long non-coding RNAs (lncRNAs), small interfering RNAs (siRNAs), piwi interacting RNAs (piRNAs), and microRNAs (miRNAs). Nevertheless, the total RNA content in sperm is extremely low, about 200 times lower than in any other cell. It is worth mentioning that in studies of the unique pool of mammalian sperm transcripts, an important step of sample preparation is the removal of somatic cells. It is assumed that RNAs of somatic cells contaminate the sample, and their presence may lead to data distortion [25]. The absence of somatic cells in sperm is a species-specific characteristic of birds, which allows for the development of faster RNA extraction protocols and can greatly facilitate sample handling [26].

The changes in RNA expression observed during spermatogenesis in birds have their own peculiarities, determined primarily by homogametic spermatozoa [27]. The first microarray study of rooster semen RNA transcripts revealed that the number of rooster genes common to sperm and testes was lower than in mammals (87.3%), while the content of “foreign” RNA, in contrast, was significantly higher (12.7%). Most of the rooster semen genes, as in mammals, were related to signal transduction, embryonic development, and cellular structure, while a smaller part, which is not typical for mammals, was related to spermatogenesis and fertilization. This fact indicates selective conservation of transcripts in bird sperm. A decrease in the activity of all genes related to ribosomal mechanisms was also noted, which, according to the authors, indicates a translation disorder in bird sperm [28]. According to other researchers, 2597 lncRNAs were identified in rooster testis tissues by high-precision sequencing, less than 1% of which showed significant sequence similarity with similar lncRNAs in human and mouse [29].

**Figure 5:**
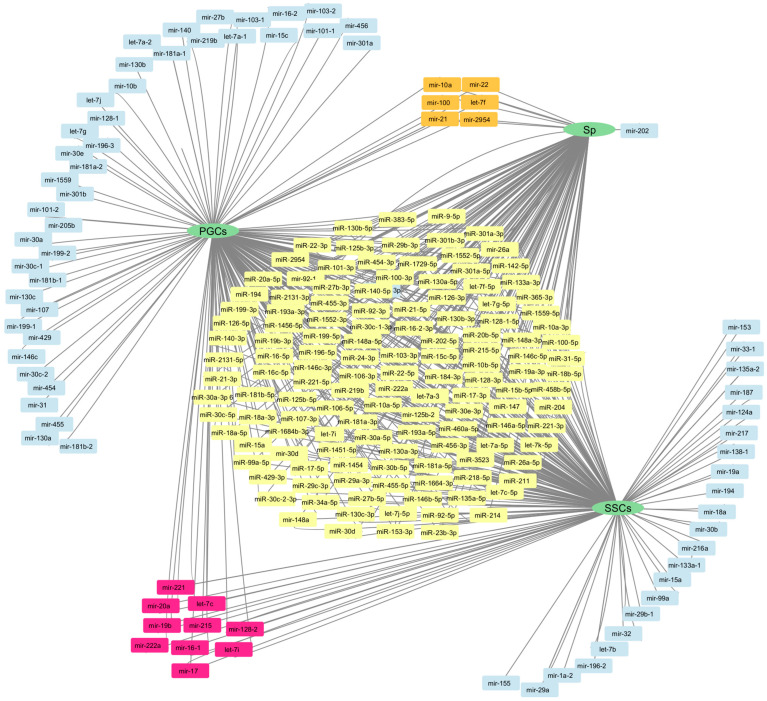
MiRNAs expressed in different types of rooster germ cells. A network of miRNAs expressed in different types of rooster germ cells. The miRNA clusters are formed by miRNAs expressed in several cell types, and the lineages lead to the cell types themselves (adapted from Xu, L. et al., 2017 [34] and Guo, Q. et al., 2021 [35]). The central yellow cluster contains miRNAs expressed in all three cell types PGCs, SSCs and Sp; pink cluster for PGCs and SSCs; orange cluster for PGCs and Sp cells (PGCs—primordial germ cells; SSCs—spermatogonial stem cells; Sp—spermatozoa). Gene and miRNA networks were visualized using the https://cytoscape.org/ (accessed on 13 December 2024) platform.

In general, spermatogenesis is strictly regulated by the neuroendocrine system [30]. and is coordinated through genetic and epigenetic mechanisms involving small RNA molecules—miRNAs. This molecule is crucial for the regulation of gene activity, which is particularly important in cellular processes such as division, differentiation, and apoptosis. Studies also show that certain miRNAs can be specifically expressed in germ cells of different development stages, emphasizing their role in ensuring the normal process of spermatogenesis (Figure 5).

For example, some miRNAs may play a protective role in preventing spermatocyte apoptosis, while others may initiate programmed cell death at a stage where it is necessary for quality selection. These complex interactions maintain a balance between spermatogenic cell proliferation and differentiation, which is critical for reproductive health [31,32]. In mammals and humans, the miRNAs involved in various steps of spermatogenesis are well studied [33]. However, in birds, this issue is still under active investigation and available data are scarce. Thus, 15 miRNAs and 21 of their target genes presumably involved in germ cell development in roosters were identified by miRNAome sequencing of three germ cell types at early stages of spermatogenesis (primordial germ cells (PGCs), spermatogonial stem cells (SSCs), and spermatogonia (Sp)). The miR-202-5p molecule and its target gene *LIMK2*, which was highly expressed in PGCs, was identified as the most significant molecule by RT-PCR and dual-luciferase reporter assay, indicating its involvement in spermatogenesis [34]. Analysis of miRNA profiles by NGS in cell cultures representing different stages of spermatogenesis revealed 128 miRNAs with differential expression. Of these, 19 were specific for PGCs, 6 for SSCs, and 4 for Sp. Among them, miRNA-301a-5p was identified as the key miRNA as it showed co-expression for all stages. Another study, in which RT-PCR, miRNA inhibition, and RNA interference methods were applied, found that miRNA-301a-5p negatively regulates the target gene *TGFβ2* by directly interacting with it [35]. *TGFβ* and TGF-beta receptor isoforms have previously been found to play important roles in testicular development and also affect steroidogenesis. A study of the effects of TGFβ isoforms on embryonic gonadal development revealed that the testes from TGFβ2 null-mutant mice are characterized by a reduced number of seminiferous cords at day 15 of embryonic development [36].

Investigation of the role of miR-31 found that it plays a negative regulatory role in the process of meiosis in roosters, and its expression is decreased during the differentiation of rooster germ cells. Expression of miR-31 is positively regulated by retinoic acid and competes with *STRA8* for retinoic acid binding. In earlier in/ex vivo experiments using rooster embryonic stem cell culture, the *STRA8* gene, a meiosis-inducer required for both female and male germ cells to enter meiosis, was found to target miR-218. Disruption of miR-218 expression resulted in decreased mRNA and protein levels of the *STRA8* gene. In mature roosters, testicular injections of miR-218 resulted in decreased semen volume [37,38].

Energy metabolism and ATP production in Sertoli cells also play an important role in spermatogenesis. A recent study found that plasma-induced apoptosis of rooster Sertoli cells in vitro shows downregulation of miR-7450 expression, which is caused by a decrease in mitochondrial metabolism and ATP production via regulation of the AMPK-mTOR signaling pathway [39].

Thus, although avian spermatogenesis proceeds similarly to mammalian, there are notable differences, including the absence of somatic cells in sperm, homogametic spermatozoa, and the uniqueness of sperm and testicular transcriptomes. All this assumes the presence of a unique miRNAome in birds. The presence of miRNAs in tissues of reproductive organs and semen opens new possibilities for understanding their role in the complex processes of male reproduction.

## 3. MiRNA Biogenesis and Its Role in Regulation of Spermatogenesis

**Figure 6:**
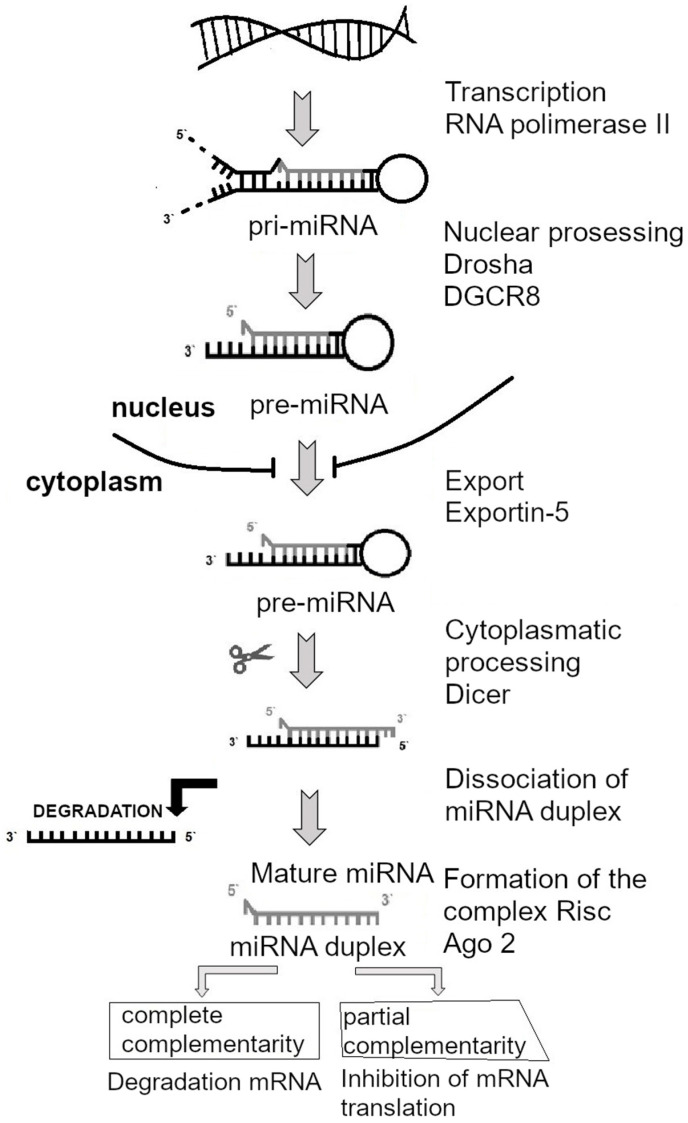
Schematic representation of microRNA biogenesis in the cell. The precursors, named pre-miRNAs, with the “stem-loop” structure are transcribed from long primary miRNAs. The primary miRNAs are then cleaved by the Drosha/DGCR8 endonuclease in the nucleus and afterwards exported to the cytoplasm by the exportin-5 transport receptor. In the cytoplasm, the pre-miRNA molecule undergoes secondary cleavage by the endonuclease Dicer, thereby becoming a mature miRNA molecule. MiRNA including one of the duplex chains is incorporated into the RISC and directed to target mRNAs, causing translational repression and decay of the target mRNA [43,44,45,46].

The 2024 Nobel Prize in Physiology or Medicine has been awarded to US scientists Victor Ambros and Gary Ruvkun for their discovery of miRNAs and their role in post-transcriptional gene regulation. MiRNA molecules are non-coding regulatory RNAs of 18–24 nucleotides in length. They were first described in nematodes *Caenorhabditis elegans* in 1993 as regulators of postembryonic development [40,41]. They are known to control up to 60% of the genome in mammals and birds [42]. MiRNA biogenesis begins in the cell nucleus (Figure 6). The precursors, named pre-miRNAs, have a structure called “stem-loop” and are transcribed from long primary miRNAs. The primary miRNA is then cleaved by the Drosha/DGCR8 endonuclease [43] in the nucleus and afterwards exported to the cytoplasm by the exportin-5 transport receptor [44]. In the cytoplasm, the pre-miRNA molecule undergoes secondary cleavage by the endonuclease Dicer, thereby becoming a mature miRNA molecule [45]. MiRNA, including one of the duplex chains, is incorporated into the RNA-induced silencing complex (RISC) and directed to target mRNAs, causing translational repression and decay of the target mRNA. A single miRNA can target multiple mRNAs, but also multiple miRNAs can target a single mRNA [42,46].

A number of studies using mouse models indicate that impaired miRNA biogenesis leads to defects at different stages of spermatogenesis [47]; however, there are practically no such studies for poultry.

The microprocessor complex of the canonical miRNA biogenesis pathway mainly consists of Drosha and DGCR8 components, which are known to have significant roles in microRNA biogenesis and male fertility formation. Dicer1 and Drosha (Stra8-Cre) knock-out mice exhibited abnormalities in the seminiferous epithelium and germ cell depletion in adult males, and the miRNAome of their spermatogenic cells significantly differed from wild-type mice [48]. Mice deficient in Dicer1 or Dgcr8 in male germ cells were infertile. They had reduced testicular weight, and histological analysis revealed a decrease in the diameter of the seminiferous tubules, along with a decrease in the number of immature cells and spermatozoa in the epididymis [49]. Although this problem has not yet been studied in birds, studies by Abu-Bonsrah et al. (2016) have shown that it is possible to modify the avian genome to investigate the effects of Dicer1 and Dgcr8. Thus, the CRISPR/Cas9 method demonstrated efficiency in genetic engineering of chicken embryos as it created gene knockouts and altered DGCR8 in neurons. Application of genetic engineering techniques revealed that DGCR8 associated genes *DROSHA*, *YPEL1*, and *NGN2* have lower expression levels after electroporation in vivo [50].

Dicer1 is an RNase III enzyme that cleaves pre-miRNAs into mature miRNAs. Inactivation of Dicer1 in the male line of PGCs in mice resulted in complete infertility and lack of functional spermatozoa due to the occurrence of multiple meiosis defects during spermatogenesis [51]. Analysis of germ cell-specific Dicer1 knock-out mice revealed a significant reduction in the number of seminiferous cords and a decrease in proliferating Sertoli cells, leading to a disruption in the development of testicular structure [52]. In Dicer1 knockout mice, spermatozoa in semen from caudal epididymis exhibited abnormal motility expressed as circular movements, as well as a flagellar defect, namely a curved flagellum [53]. Dicer splicing variants at the molecular level were studied in female geese and their role in the regulation of follicle maturation was identified. Thus, gDicer is required for follicle growth, and the different functions of the two gDicer isoforms in the regulation of granulosa cell death may be related to structural changes in their helicase domains [54]. Given the key role of Dicer enzyme in miRNA biogenesis, it can be assumed that it is potentially important for the regulation of reproductive processes in both sexes.

The significant role of Argonaute effector proteins (AGO) has also been shown. The Argonaute family includes eight proteins, which are classified into two subfamilies: EIF2C/hAGO1 (four members) and PIWI (four members). The specific feature of AGO family proteins is the presence of two domains: PAZ and PIWI [55]. Similar to DICER proteins, the PAZ domain of AGO proteins interacts with 3′ 2-nt overhangs of siRNA and miRNA. The PIWI domain functions as a binding site for RNA strands [56].

AGO2 protein is found in both cytoplasmic and nuclear compartments. In the nucleus, this protein binds both chromatin and nucleus-specific mRNA transcripts of hundreds of genes, which are crucial for sperm production. Also, in the germline, conditional knockout of AGO2 causes depletion of the corresponding proteins and defects in sperm number and morphology [57]. Ago2 knockout mice exhibited decreased total sperm count, abnormal head morphology of spermatozoa, and decreased postnatal offspring viability [58].

Several studies on the biological functions of the PIWI subfamily genes confirm their key role in meiosis during rooster spermatogenesis. For instance, the expression level of the cloned Piwi-like 1 (*PIWIL1*) gene varied and was detected at a low level in embryonic stem cells such as PGCs and SSCs. As meiosis progressed, the expression level of *PIWIL1* increased, reaching maximum values at the later stages of meiosis [59,60]. The role of PIWIL1 protein and its Piwi/Argonaute/Zwille (PAZ) domain in chicken embryonic development was investigated in the study by Qi-Xin et al. (2023). Injection of the pCMV-Cas9-puro-sgRNA-2 genetic construct into two-day-old embryos resulted in 19 PAZ mutants (13 males and 6 females). These mutants exhibited delayed hatching, decreased semen quality, and decreased expression of *PIWIL1* and *SOX2* genes on days 5 and 18 of embryonic development, confirming the negative effect of PAZ mutation on semen quality parameters in roosters [61].

The aforementioned studies prove that miRNA biogenesis is a complex, multistep process and its role in spermatogenesis is undoubted, yet not fully explored. In addition, these data indicate the importance of further study of miRNA mechanisms of action for successful realization of their potential in poultry farming.

## 4. Association of microRNA with Sexual Dimorphism in Birds

Gender determination and differentiation are complex biological processes that determine the development of reproductive organs and secondary sex characteristics. During these processes, the bipotential gonad (undifferentiated testicular or ovarian precursor) acquires the fate of either testicular or ovarian cells, and secondary sex characteristics develop according to a male or female model. In birds, the sex-determination system is genetically programmed and regulated by miRNAs [62], which play a critical role in various developmental processes of the mammalian reproductive system at the embryonic stage [16]. Primordial germ cells (PGCs) are the initial population of germ cells formed during the early stages of embryogenesis. They serve as precursors to both oogonia and spermatogonia. A number of studies indicate the essential role of sexually dimorphic miRNAs in the processes of sex development. Sequencing of both right and left gonads in 5.5-day-old embryos revealed 7 sex-linked miRNAs in female and male gonads (miR-1416-5p, miR-204, miR-211, miR-2954, miR-3538, miR-6606-5p, miR-7b), among which miR-7b, miR-211 miR-204, and miR-302b-5p are hypothesized to play crucial roles in ovarian development [63]. Using Solexa sequencing followed by qRT-PCR verification, the researchers analyzed miRNA expression in the gonads of embryos at the 5.5-day stage. The results showed that miR-107 specifically regulates ovarian development through post-transcriptional modification of *NR5A1* and *CYP19A1* genes, which are involved in estrogen signaling pathways [64]. Dual-luciferase reporter assay in DF1, overexpression and inhibition of miR-92 in chicken embryonic fibroblasts (5.5 days old) showed that miR-92 has a parallel role in chicken gonadogenesis by regulating *ATRX* and *DDX3X*, which encode proteins essential for Sertoli cell development. Notably, these genes are highly conserved and in mammals are located on the X chromosome, whereas in birds they are located on autosomes [65]. Sequencing method identified 113 miRNAs that showed different degrees of expression in chicken embryonic stem cells before and after their differentiation into male germ cells. Among them, miRNA-383-5p was identified as statistically significant. Its target gene *HDAC9* was found to positively regulate the differentiation process of chicken embryonic stem cells [66,67]. In another study, Solexa sequencing identified miR-2954 as a significant miRNA, and application of in vivo-morpholino injection established its role in sex differentiation processes in Jingfeng No.1 chicken embryos. Increased expression of miR-2954 was observed in ZW gonads, and its reduction affected the expression of *DMRT1* and *SOX9* genes, which are involved in the formation of male gonads [68]. Further studies showed that miR-2954 is linked to the Z chromosome and plays a key role in sex-chromosome dosage compensation. Knockout of miR-2954 results in the death of male embryos [69].

Recently, the role of miRNAs has become increasingly important in the study of stem cell pluripotency. The results of in vitro studies performed on chicken embryonic stem cell cultures indicate that inhibiting the expression of target genes of miR-302b-5P leads to a decrease in both proliferative activity and the frequency of cell apoptosis [70,71].

It is worth mentioning that the influence of miRNAs on sex determination in birds is a complex issue and requires further study. These molecules play an important role in the regulation of gene expression and influence signaling pathways involved in the development and formation of gonads. Further research work in this direction will lead to a better understanding of the role of microRNAs in the realization of sexual dimorphism in birds.

## 5. Association of microRNA with Sperm Quality in Birds

Sperm quality in roosters is the key factor in successful insemination and healthy offspring. The ability to move in a straightforward direction is formed during the development of the spermatozoon, and miRNAs play an important role in regulating this process at the molecular level. The mRNA and miRNA transcriptomes of epididymis tissues from roosters with low and high sperm motility were comparatively analyzed using high-throughput sequencing. As a result, 84 genes and 6 miRNAs (mir-146a, miR-215, miR-194, miR-135b, mir-205b, mir-214) showing differential expression between the two groups analyzed were identified. Among them, 3 miRNAs (miR-146a, mir-135b, and mir-205b) are hypothesized to play important roles in the regulation of sperm maturation, capacitation, and motility. The genes *MMP9*, *SLN*, *WT1*, *PLIN1*, and *LRRIQ1* were considered as the most promising candidates potentially affecting sperm motility in the epididymis of roosters [72].

In another study, sequencing of testicular tissue miRNAomes in groups of Beijing-You roosters with different levels of sperm motility revealed 23 differentially expressed miRNAs. Only miR-155, miR-7480-5p, and their target genes (*KCNA1* and *AHI1*, respectively) were found to be statistically significant [73]. According to other authors, the testicular tissue transcriptome of Jing Hong No.1 roosters contained 15 differentially expressed miRNAs, and the regulatory pathway including MSTRG.3077.3/MSTRG.9085.1-gga-miR-138-5p-CADM1 and MSTRG.2290.1-gga-miR-142-3p-GNAQ/PPP3CA was identified as crucial in modulating sperm motility [74].

microRNAome profiling of extracellular vesicles of Beijing-You roosters seminal plasma revealed 1006 miRNAs in groups of individuals with high sperm motility, and 1084 miRNAs in groups with low motility, of which 34 miRNAs showed different expression profile in the analyzed groups. The miR-24-3p has been identified as one of the significant miRNAs for the establishment of fertility in male roosters [75]. Previously, 1077 miRNAs (851 known and 226 novel) were detected in the extracellular vesicles of seminal plasma in studies conducted on 4 rooster breeds (Beijing-You, Dwarf, Recessive White, and White Leghorn). MiRNAs such as miR-10b-5p, miR-10a-5p, and miR-100-5p were identified as the most frequently expressed in all breeds. The results obtained may indicate some breed specificity of miRNAs [76].

Several studies provide information about the effects that certain miRNAs have on rooster semen quality (Table 1). Intensive study of the expression and functional characteristics of miRNAs in birds reveals their critical significance in the regulation of spermatogenesis and, consequently, fertility.

Thus, studying the functions of miRNAs in avian semen may have significant practical applications. Controlling the expression of specific miRNAs can improve semen quality and reproductive performance, and analyzing expression changes of miRNAs in semen can be used for early detection of fertility problems in birds.

## 6. Conclusions and Perspectives

To date, recent studies have provided some information on the role of miRNAs in biological processes related to fertility of male birds. By performing a fine regulation of genes at the posttranscriptional level, miRNAs determine the success of biological processes that ensure fertility of males starting from early embryonic development and throughout the life of the individual. Of particular note are those issues of miRNA biogenesis that highlight the important role of these molecules in the development of male fertility. However, the functional role of miRNAs in male reproduction and the underlying molecular mechanisms remain insufficiently studied. On the one hand, this limits the application of miRNAs as biomarkers in poultry production, but, on the other hand, it opens new horizons for research strategies and defines future perspectives [84,85].

Improved gene-editing technologies in birds, such as CRISPR/Cas9, allow the application of targeted mutagenesis to study the role of individual genes [50]. NGS technologies have significantly impacted the field of miRNA research. The transition from traditional Sanger sequencing to NGS methods, as well as third-generation sequencing technologies, has improved the efficiency of studying miRNA expression profiles [86]. The existing NGS software (MiREvo is standalone, modular, and freely available at http://evolution.sysu.edu.cn/software/mirevo.htm (accessed on 18 December 2024) under the GNU/GPL license) platforms provide detailed miRNA sequencing and analysis, allowing researchers to identify and characterize these small non-coding RNAs [87]. Since there are challenges in identifying miRNAs due to their small size, specific distribution, and high instability, a next-generation sequencing technology, Roche 454 GS-FLX, was developed to avoid cloning steps in bacteria and provide a fast and accurate tool for genome-wide detection of novel miRNAs and other mRNAs [88]. In addition, recent advances in bioinformatics have greatly improved the accuracy and efficiency of data integration in miRNA, dnRNA, and mRNA libraries. One of the approaches of interest, namely DeepLPI, is a multimodal method developed to predict interactions between long non-coding RNAs and protein isoforms. This method accounts for the complexity of gene coding and isoform interactions and allows for the fact that a single gene can encode multiple protein isoforms, whereas traditional computational methods often miss this feature [89].

Integrating miRNA studies with other -omics technologies can provide a more complete understanding of gene regulation and mechanisms of fertility impairment in male birds. By combining miRNA data with data from genomics, transcriptomics, proteomics, and metabolomics, researchers can uncover new insights into the complex biological processes of avian reproduction.

The development of targeted strategies to manipulate miRNA expression is crucial for understanding their role in various biological processes. These strategies may include the use of synthetic oligonucleotides, small-molecule inhibitors, or gene-editing technologies to modulate miRNA levels in a specific and controlled manner. By identifying specific miRNAs whose expression is impaired in pathological conditions, researchers can potentially develop targeted therapies for the pathologies under investigation.

The results obtained should be translated into practical applications to improve fertility and productivity of roosters. Such practical applications should be based on the development of specialized treatments or breeding solutions to improve the reproductive performance of poultry. Understanding the mechanisms by which certain miRNAs affect rooster fertility may help researchers control these molecules and, hence, to increase the efficiency of the poultry industry.

Addressing the specific problems of impaired rooster fertility through studies of miRNA mechanisms requires the development of new approaches and strategies. MiRNA as a research subject is gaining popularity in the area of agriculture, with a particular focus on fertility enhancement methods in poultry.

## 7. Search Strategy and Inclusion Criteria

The present non-systematic overview provides an analysis of the current data on the role of miRNAs in spermatogenesis of male birds. The manuscript is based on a selective study of high-quality articles covering the current state of research on the functional role of miRNAs in the regulation of spermatogenesis, sex differentiation, and morphofunctional characteristics of avian spermatozoa. The issues of miRNA biogenesis in the aspect of male reproduction are also considered. Reference sources were searched for in 2024. No publication date relevance requirement was noted for the sources used in the review. The main objective was to identify trends and increase understanding of the current state of research in this field. Relevant references were collected from PubMed, Google Scholar, and ResearchGate. Terms such as “miRNA”, “spermatogenesis”, “semen”, “reproduction”, “testis”, “sperm motility”, “birds”, “chick”, “poultry”, “biogenesis”, “sex differentiation”, “transcriptome”, “gene”, “expression” and “heme cells” were used in the search. Additional articles were found by analyzing bibliographies of relevant publications, and only studies in English were considered.

## Figures and Tables

**Table 1:** Summary of microRNAs and their target genes associated with avian spermatogenesis.

miRNA	Target Gene	Function of the Target Gene	Avian Species	Cell Type	Cellular Processes	References
oha-miR-182-5p	*LOC106049708* (TRAILR1)	expressed in Sertoli cells and mature germ cells; participates in maintaining testicular homeostasis, promoting the elimination of damaged germ cells and regulating the number of developing cells in accordance with the capacity of Sertoli cells	Goose	testicular tissue	miR-182-5p is involved in the regulation of apoptotic pathways during spermatogenesis and may enhance testicular microenvironment stability and sperm quality by targeting LOC106049708	Qin S.B. et al., 2018 [77], Chen K.Q. et al., 2022 [78] Hao H. et al., 2024 [79]
ocu-miR-10b-5p	*PI3K/AKT*	*PI3K/AKT* signaling may promote proliferation and anti-apoptosis of immature Sertoli cells and spermatogenic cells			miR-10b-5p is involved in the regulation of *PI3K/AKT* signaling, playing an important role in maintaining homeostasis of ATP and glucose metabolism. A decreased expression of miR-10b-5p was observed in the low sperm motility group of males	
miR-140-3p let-7d miR-145-5p	*NKAIN3*	transport of Na^+^ and K^+^ ions and energy metabolism in cells; expressed in chicken sperm membranes, participating in fertilization	Goose	testicular tissue	miR-140-3p; let-7d; miR-145-5p regulate the expression of genes associated with sperm motility.	Wu Q. et al., 2023 [80]
	*BTG1*	expressed in meiotic and early meiotic germ cells				
	*CLEC2E*	may prevent pathogen invasion during sperm formation (protective function)				
	*LINC9137*	regulation of Sertoli cell proliferation and apoptosis in the testicles	Goose	testicles	*LINC9137* regulates goose testicular Sertoli cell proliferation and apoptosis via miR-140-3p overexpression of miR-140-3p significantly suppressed the expression of *LINC9137* and *NKAIN3* in Sertoli cells, and their expression was significantly increased when miRNA expression was disrupted	Yingping W. et al., 2024 [81]
miR-140-3p	*NKAIN3*	sodium/potassium ATPase-interacting transporter protein				
miR-183-5p	*FOXO1*	the major regulator of stem cell self-renewal and differentiation; regulates multiple aspects of spermatogenesis, ranging from long-term stem cell self-renewal to the initiation of spermatogenesis and meiosis; overexpression results in increased sperm motility.	Domestic pigeon	testicles	miR-183 expression was reduced in the high sperm motility group with increased *FOXO1* expression	Johnson A.R. et al., 2010 [82] Yin Z. et al., 2021 [83]
miR-32-5p	*CHDH*	the main enzyme involved in choline metabolism catalyzes the oxidation of choline to betaine in the inner mitochondrial membrane, which is an important organelle influencing sperm maturation			the expression of *CHDH*, which is a target gene of miR-32-5p, was increased in the high sperm motility group.	
miR-301a-5p	*TGFB2*	TGFb isoforms and TGF-beta receptor isoforms control testicular development and influence steroidogenesis, tubule formation, and gonocyte behavior	Rooster sperm cell culture (in vitro)	primordial germ cells (PGCs), spermatogonial stem cells (SSCs), spermatogonia (Spa) in semen	miR-301a-5p negatively regulates *TGFB2* through direct binding	Guo Q. et al., 2021 [35]
miR-31	*ADAM10*	apoptosis and germ cell differentiation	Rooster	testicles	miR-31 plays a negative regulatory role during meiosis in rooster germ cells, and its expression is decreased during rooster germ cell differentiation. miR-31 transcription is positively regulated by retinoic acid and competes with *STRA8* for retinoic acid binding	Wang et al., 2023 [37]
miR-218	*STRA8*	meiosis inducer required for both female and male germ cells to enter meiosis	Rooster	testicles	miR-218 overexpression suppresses *STRA8* expression, resulting in decreased sperm volume.	Wang et al., 2020 [38]
miR-7450	*AMPKα*	regulates intracellular ATP levels through activation of mitochondrial biogenesis, and inhibition of respiratory chain enzymes	Rooster somatic cell culture (in vitro)	Sertoli cells	decreased expression level of miR-7450 leads to increased *AMPKα* mRNA levels and activates *AMPKα* phosphorylation in chicken Sertoli cells upon exposure to non-thermal plasma	Zhang J.J. et al., 2018 [39]
miR-135b	*HPS5*	regulates the synthesis and function of lysosomes as well as other highly specialized organelles	Rooster	epididymis	expression levels of miR-135b and *HPS5* gene are negatively correlated in epididymis tissues of roosters with different sperm motility	Xing K. et al., 2022 [72]
miR-205b	*CENPO*	encodes a component of the interphase centromere complex. The protein is localized in the centromere throughout the cell cycle and is required for bipolar spindle assembly, chromosome segregation, and checkpoint signaling during mitosis.			expression levels of miR-205b and *CENPO* gene are negatively correlated in epididymis tissues of roosters with different sperm motility	
miR-155	*KCNA1*	encodes a voltage-gated delayed potassium channel	Rooster	testicles	miRNAs are thought to be involved in the activation of mechanisms responsible for sperm motility by suppressing the expression of the target gene	Liu Y. et al., 2018 [73]
miR-7480-5p	*AHI1*	involved in vesicle trafficking and required for ciliogenesis				
miR-138-5p	*CADM1*	involved in the regulation of apoptosis, cell adhesion, cell differentiation, and spermatogenesis	Rooster	testicles	involved in key pathways responsible for the regulation of sperm motility by regulating the expression of target genes	Guo S. et al., 2023 [74]
gga-miR-142-3p	*GNAQ*	involved in the binding of GTP, magnesium, metals, and nucleotides				
	*PPP3CA*	regulates ATPase binding activity, calmodulin and calmodulin-dependent protein phosphatase activity. Participates in calcineurin-NFAT signaling cascade, peptidyl-serine dephosphorylation, and calcium ion response				
miR-10a-5p miR-10b-5p miR-100-5p	*-*	-	Rooster	extracellular vesicles of seminal plasma	high expression levels in roosters of 4 breeds (Beijing-You, Dwarf, Recessive White and White Leghorn) suggest potential involvement of these miRNAs in the mechanisms of sperm maturation, capacitation, storage, and fertility.	Han X. et al., [76]

## Data Availability

Not applicable.

## Abbreviations

Abbreviations	Definitions
AGO	Argonaute effector proteins
HPG	hormones of the hypotholamic-pituitary-gonadal
ISH	in situ hybridization
lncRNA	long non-coding RNA
miRNA	microRNA
ncRNA	non-coding RNA
NGS	next-generation sequencing
PAZ	Piwi/Argonaute/Zwille
PCR	polymerase chain reaction
PGCs	primordial germ cells
piRNA	piwi interacting RNA
qPCR	real-time quantitative PCR
RISC	RNA-induced silencing complex
RT-PCR	real-time polymerase chain reaction
siRNA	small interfering RNA
Sp	Spermatozoa
SSCs	spermatogonial stem cells
